# Iron metabolism abnormalities in autoimmune hemolytic anemia and *Jianpishengxue keli* can ameliorate hemolysis and improve iron metabolism in AIHA mouse models

**DOI:** 10.1080/07853890.2022.2157475

**Published:** 2022-12-28

**Authors:** Manjun Zhao, Yan Wang, Jin Yang, Yi Wang, Yingying Feng, Lei Chen, Zonghong Shao, Huaquan Wang, Limin Xing

**Affiliations:** a Department of Hematology, General Hospital of Tianjin Medical University, Tianjin, China; b Third Medical Center of Chinese People’s Liberation Army General Hospital, Beijing, China

**Keywords:** Autoimmune hemolytic anemia, iron metabolic, hepcidin, immune inflammation, *Jianpishengxue Keli*

## Abstract

**Objective:**

Autoimmune hemolytic anemia (AIHA) is rare heterogeneous disorder characterized by red blood cell (RBC) destruction via auto-antibodies, and after RBC is destroyed, proinflammatory danger-associated molecular patterns including extracellular hemoglobin, heme, and iron which causing cell injury. And oxidative stress represents one of the most significant effects of chronic hemolysis. *Jianpishengxue keli* can improve the symptoms of anemia patients with kidney disease and tumors and are beneficial in promoting recovery from chronic inflammation. Therefore, it is presumed that *Jianpishengxue keli* can improve the symptoms of AIHA. We aimed to investigate iron metabolism in AIHA and effects of *Jianpishengxue keli* on AIHA murine model.

**Methods:**

Nineteen hemolytic episode AIHA patients, 10 remission patients and 10 healthy controls (HCs) were enrolled in this study. Serum hepcidin, ferritin and other related indicators of iron metabolism were measured. Mouse models of AIHA were established and received high, medium, or low doses of *Jianpishengxue keli* by gavage daily for 14 and 28 days respectively. The level of RBCs, Hb, bilirubin, LDH, hepcidin, and the expression level of hepcidin mRNA, and hepatic ferroportin 1(FPN1) protein were evaluated.

**Results:**

Serum hepcidin in hemolytic episode AIHA patients and remission patients were significantly higher than that in HCs (*p =* 0.0083 and *p* = 0.0473, respectively). Serum ferritin in hemolytic AIHA patients was significantly higher than that in HCs (*p =* 0.008). Serum transferrin saturation levels are increased in patients with AIHA[ (57.21 ± 8.96) %]. EPO in hemolytic group was higher than that in healthy control (*p*＜0.05). In AIHA mouse models, IBIL decreased after 14 days of high dose drug intervention. After 28 days, TBIL and IBIL both significantly decreased in all dose groups and LDH significantly decreased in the medium-and high-dose groups. Body weight improved, and the level of RBCs, Hb and hepcidin in the high-dose group returned to normal. After 14 and 28 days of intervention, hepatic hepcidin mRNA in all dose group significantly decreased. Hepatic FPN1 protein which were significantly lower in the AIHA mouse models, increased in all dose groups after drug intervention for 28 days.

**Conclusion:**

Iron metabolism abnormalities exists in AIHA patients and *Jianpishengxue keli* can ameliorate hemolysis and improve iron metabolism in AIHA mouse models.

**KEY MESSAGES**
- Iron metabolism abnormalities exists in hemolytic episode AIHA patients. Hepcidin and ferritin levels significantly elevated and also correlated with the severity of AIHA patients. *Jianpishengxue keli* can ameliorate hemolysis and prompt the recovery of AIHA.

## 1. Introduction

AIHA is a predominant form of acquired hemolytic anemia that is mediated by the immune system. Autoantibodies against red blood cells (RBCs) produced and secreted by hyperactive B lymphocytes with or without complement system activation, lead to hemolysis [1].

Inflammation may develop as a result of immune dysregulation in autoimmune diseases. Cytokines, including tumor necrosis factor α (TNF-α), interleukin-1, interleukin-6, and interferon-γ, are produced by inflammatory cells within the first hours after the onset of inflammation; these cytokines restrict erythropoiesis both directly and indirectly and shorten the erythrocyte lifespan [2]. The capacity of BFU-E to give rise to more differentiated erythroid cells is inhibited by inflammatory cytokines [3–5]. In the event of acute hemolysis, iron released from erythrocyte destruction aggravates the clinical symptoms of patients with AIHA. RBCs release proinflammatory danger associated molecular patterns (DAMPs) including extracellular hemoglobin, heme, and iron [6]. Iron ions, derived from disintegrated RBCs during hemolysis, in coagulation through FVIII/FIX interactions involving oxidative reactions [7]. Heme iron species oxidize organic compounds in the presence of hydrogen peroxide [8]. Acute hemolysis leads to the release of intraerythrocytic components into the circulation exacerbating the inflammatory response. The relapse rate of AIHA is high, and patients who are resistant to first-line treatment with glucocorticoids develop chronic disease [9]. Patients in a chronic hemolytic state have continued destruction of red blood cells, and components within the red blood cells are constantly being released, resulting in a persistent inflammatory response. Inflammation can inhibit erythropoiesis, which disrupts iron metabolism in patients with refractory AIHA [10].

*Jianpishengxue keli* is a compound preparation that includes *Codonopsis pilosula*, *Poria cocos*, *Atractylodes*, licorice, *Astragalus*, Chinese yam, *Chicken gizzard membrane*, *tortoise shell*, *Ophiopogon japonicus*, *Kadsura longepedunculata*, *oysters*, *Ziziphus jujuba*, ferrous sulfate and vitamin C [11]. It has been safely applied to treat pregnant women [12], children [13], elderly [14], perioperative [15], tumor chemotherapy [16], and various types of anemia [17]. *Jianpishengxue keli*, can improve absorption function, promote nutrient intake and absorption, and improve the hematopoietic environment and function by invigorating the spleen and stomach, and nourishing ‘*qi*’ and blood [18]. Maybe *Jianpishengxue keli* can be used to treat AIHA and improve the clinical symptoms of patients. Therefore, it is presumed that the *Jianpishengxue keli* can be used to treat AIHA.

We firstly analyzed the iron indexes in AIHA patients, and then established AIHA mouse model and administered *Jianpishengxue keli* to mouse by gavage, and observed the changes in AIHA models to understand the effect of *Jianpishengxue keli*.

## 2. Materials and methods

All study participants provided written informed consent, and the study design was approved by the Ethics Committee of Tianjin Medical University General Hospital and performed in accordance with the Declaration of Helsinki. Animal experiments involved in this study were performed adhered to Chinese Small Animal Protection Association Experimental Protocol.

### 2.1. Patients

Twenty-nine AIHA patients who were admitted to the Department of Hematology of Tianjin Medical University General Hospital from May 2020 to December 2020 were enrolled (19 hemolytic episode AIHA patients, including 13 females and 6 males whose median age were 64 years old; 10 remission AIHA patients including 7 females and 3 males whose median age were 60 years old). There were 10 healthy controls (HCs), including 8 females and 2 males whose median age were 47.5 years old. All AIHA patients were diagnosed in accordance with the *2017 Chinese expert consensus* [19]. The criteria for the diagnosis of AIHA were as follows: The level of hemoglobin (Hb) should meet the diagnostic criteria for anemia (Male < 12g/dL; Female < 11g/dL); RBC autoantibodies should be detected in the patients; the results of laboratory tests should meet at least one of either: Percentage of reticulocytes (Ret%) >4% or absolute value >120 × 10^9^/L; haptoglobin (Hp) <100 mg/L; and total bilirubin (TBIL)≥17.1 mol/L, which Indirect bilirubin (IBIL) is mainly elevated. If the patients showed a good response to glucocorticoid or even if the Coombs test is negative, AIHA should also be diagnosed. The exclusion criteria included serious organ dysfunctions, uncontrollable active infection, human immunodeficiency virus positivity, pre-existing known malignancy, pregnancy, other autoimmune diseases and unwillingness to comply with the protocol.

The standard of remission refers to an increase in hemoglobin > 2 g/dL or normalization of hemoglobin, hemolysis without biochemical regression; and no blood transfusions in the last 7 days [1].

### 2.2. Mouse models of AIHA

C57BL/6J (B6) mice were purchased from the Beijing Vital River Laboratory Animal Technology Co. Ltd. Female C57BL/6J (B6) mice (20–25 g) were housed in a temperature-controlled environment at 23–25 °C with a 12/12 h light/dark cycle and were given access to food and water without restriction. All care and handling of mice were performed according to the standard guidelines for the care and use of experimental animals in Chinese Academy of Medical Sciences&Peking Union Medical College Institute of Radiation Medicine.

### 2.3. Immunization regimen for induction of AIHA

Rat RBCs was purchased from Zhengzhou Bestgene biotech company (Henan, China). Sixty-five mice were selected and randomly divided into normal control group, model group. We established AIHA mouse models using classical rat erythrocyte intraperitoneal immunization [20,21]. After adaptive feeding for one-week, healthy female C57BL/6J mice (age, 6–8 weeks; weight, 20–25 g) were weighed, then blood was collected from the inner canthus. Routine blood parameters were evaluated, and plasma levels of TBIL, DBIL, IBIL, LDH, and hepcidin were measured. Stock suspensions of rat RBCs (1 × 10^9^/mL of normal saline) were prepared, then the experimental group was injected intraperitoneally (i.p.) with 2 × 10^8^ RBCs/200 μL of normal saline, and the controls were injected i.p. with 200 μL of normal saline on the first and third days of each week for 12 weeks. The overall status of the mice was assessed, and they were weighed weekly. Routine blood parameters were evaluated, and plasma levels of TBIL, direct bilirubin (DBIL), IBIL, lactate dehydrogenase (LDH), and hepcidin were measured at 12 weeks. Normocytic normochromic anemia or Hb levels decreased by > 2 g/dL, and increased plasma levels of bilirubin and LDH indicated successful modeling.

### 2.4. Jianpishengxue keli

*Jianpishengxue keli impregnation* were gifted by Wuhan Jianmin Co. Based on the clinical dosage of *Jianpishengxue keli*, the adult dosage is 45 g/day according to drug instructions, which is equivalent to 0.64 g/kg at 70 kg, and the general formula of body surface area lgS = 0.8762 + 0.698lgW converts the equivalent dosage of *Jianpishengxue keli* for rats to about 3.5 g/kg. In this experiment, the low dose was 3 times the clinical dosage, the middle dose was 9 times the clinical dosage, and the high dose was 27 times the clinical dosage. 0.11 kg of *impregnation* was required for each 1 kg of granules, and the conversion from granules to *impregnation* resulted in low, middle, and high doses of 2.9, 5.8, and 8.7 g/kg, respectively [22].

### 2.5. Methods

#### 2.5.1. Hepcidin and ferritin evaluation in patients with AIHA

Peripheral venous blood (5 mL) extracted from the patients and healthy controls was centrifuged at 1,500 rpm for 10 min, then hepcidin and ferritin levels were respectively determined in supernatants using an ELISA (Wuhan Colorful Gene Biological Technology Co., Ltd) and radioimmunoassay kits (Elabscience). Correlations with other clinical hemolytic and immunological indicators were also analyzed.

#### 2.5.2. Intervention with Jianpishengxue keli in AIHA mouse models

The models were randomly placed into four cages (*n* = 10 each) then randomly assigned to receive, 2.9 (low), 5.8 (medium), or 8.7 (high) g/Kg/d of *Jianpishengxue keli* via gavage. The untreated group was given an equal volume of pure water as a negative control. The mice were weighed on days 14 and 28 after the first administration. Blood was collected from the inner canthus vein. Changes of the indices including red blood cell count (RBC), hemoglobin (Hb) and hematocrit (HCT) were determined by Advia 120 Hematology System (Bayer, Tarrytown, NY). TBIL, DBIL, IBIL, LDH were measured by Beijing BoHeng Kechuang Biological science and technology Co., Ltd. Hepcidin were measured using ELISA Kit (Shanghai Ucove Biotechnology Co.).

#### 2.5.3. Hepatic hepcidin mRNA detection by qRT-PCR

The mice sacrificed by anesthesia using CO2. Total RNA from mouse livers was extracted using the TRIzol reagent. Concentrations of RNA and A260/A280 ratios were determined by spectrophotometry. The extracted RNA was reverse transcribed according to the manufacturer’s instructions. Complementary DNA was synthesized from 1 μg of total RNA using AccuPower RT premix kits (BIONEER, Korea). RT-PCR analysis was performed using the TB GreenTM Premix Ex Taq II kit (TAKARA, Japan) and iQ5 real-time PCR system (Bio-Rad Laboratories Inc., Hercules, CA, USA) following the manufacturer’s instructions. Each sample was analyzed in triplicate, and the target genes were normalized to the reference housekeeping gene *gapdh*. The reaction conditions comprised pre-denaturation at 95 °C for 10 min, followed by 50 cycles of 5 s at 95 °C and 45 s at 55 °C. Table 1 showed the PCR primer sequences which were synthesized by Genechem Co., Ltd (China). The results were calculated using the conventional 2^-△△Ct^ method.

**Table 1.** Primer sequences for fluorescence quantitative PCR.

Primer sequences for fluorescence quantitative PCR	
Primer	Sequences (5′–3′)
Hepcidin	F- TGCCTGTCTCCTGCTTCTCCTC
	R- AATGTCTGCCCTGCTTTCTTCCC
GAPDH	F-CCCTTAAGAGGGATGCTGCC
	R-ACTGTGCCGTTGAATTTGCC

#### 2.5.4. Hepatic FPN1 detection by Western blotting

The mice sacrificed by anesthesia using CO2. Total protein was extracted from mouse liver using protein lysate (RIPA: PMSF = 100:1). The total protein was determined by the BCA protein assay kit (Thermo). Proteins in samples were fully denatured by heating in 4× loading buffer at 100 °C for 10 min, separated by sodium dodecyl sulfate polyacrylamide gel electrophoresis and transferred to polyvinylidene difluoride membranes (Millipore, Billerica, USA). Membranes were blocked with non-fat milk and probed with primary antibodies (anti-TfR1, anti-FPN1, and anti-GAPDH) (Santa Cruz). Membranes were then incubated with horseradish peroxidase-conjugated anti-rabbit IgG (Santa Cruz). After washes three times the blots were visualized by enhanced chemiluminescence with chemiluminescence system machine (Clinx Company, ChemiScope 6300). Gray value of Western blot strips was quantified with Image J software (National Institutes of Health).

#### 2.5.5. Statistical analysis

The results were expressed as the mean ± standard deviation. Comparisons between two groups were analyzed with Student’s *t-*test. For several independent groups, one-way ANOVA was used. Pearson correlation test was adopted for correlation analysis. *p* Values of less than 0.05 were considered statistically significant. All analyses were performed by SPSS 22.0 software. All the figures and graphs were made using GraphPad Prism 8 software.

## 3. Results

### 3.1. The characteristics of AIHA patients

Characteristics of the AIHA patients enrolled in this study are shown in Table 2. The patients and healthy controls were of comparable age and were all middle-aged. All patients were warm antibody AIHA and none had undergone splenectomy. Some patients in the hemolytic episode group had red blood cell transfusions.

**Table 2.** Clinical characteristics of AIHA patients.

	Hemolytic group	Remission group	Healthy controls
Number	19	10	10
Age	64 ± 11	60 ± 19	58 ± 12
Gender (female/male)	13/6	7/3	6/4
Hb (g/dL)	7.7 ± 1.60	9.2 ± 2.68*	12.0 ± 1.67
Type	Warm Ab	Warm Ab	–
RET (%)	14.22 ± 2.091	3.22 ± 1.01	1.50 ± 0.11
Hepcidin (pg/ml)	27704.13 ± 19814.80**	20247.41 ± 10277.51*	9482 ± 3572
Ferritin (ng/ml)	367.15 ± 537.41**	313.89 ± 576.38*	20.06 ± 57.33
Transferrin saturation (%)	57.21 ± 8.96*	40.22 ± 3.16	30.23 ± 10.2
EPO (mIU/ml)	265.7 ± 80.08*	105.2 ± 30.1*	19.16 ± 5.33
Red blood cell transfusions(U)	0.84 ± 1.21	0	0
Splenectomy	No	No	–

Compared with normal control group, *p<0.05; **p<0.01.

### 3.2. Serum hepcidin and ferritin levels in patients with AIHA

**Figure 1.**
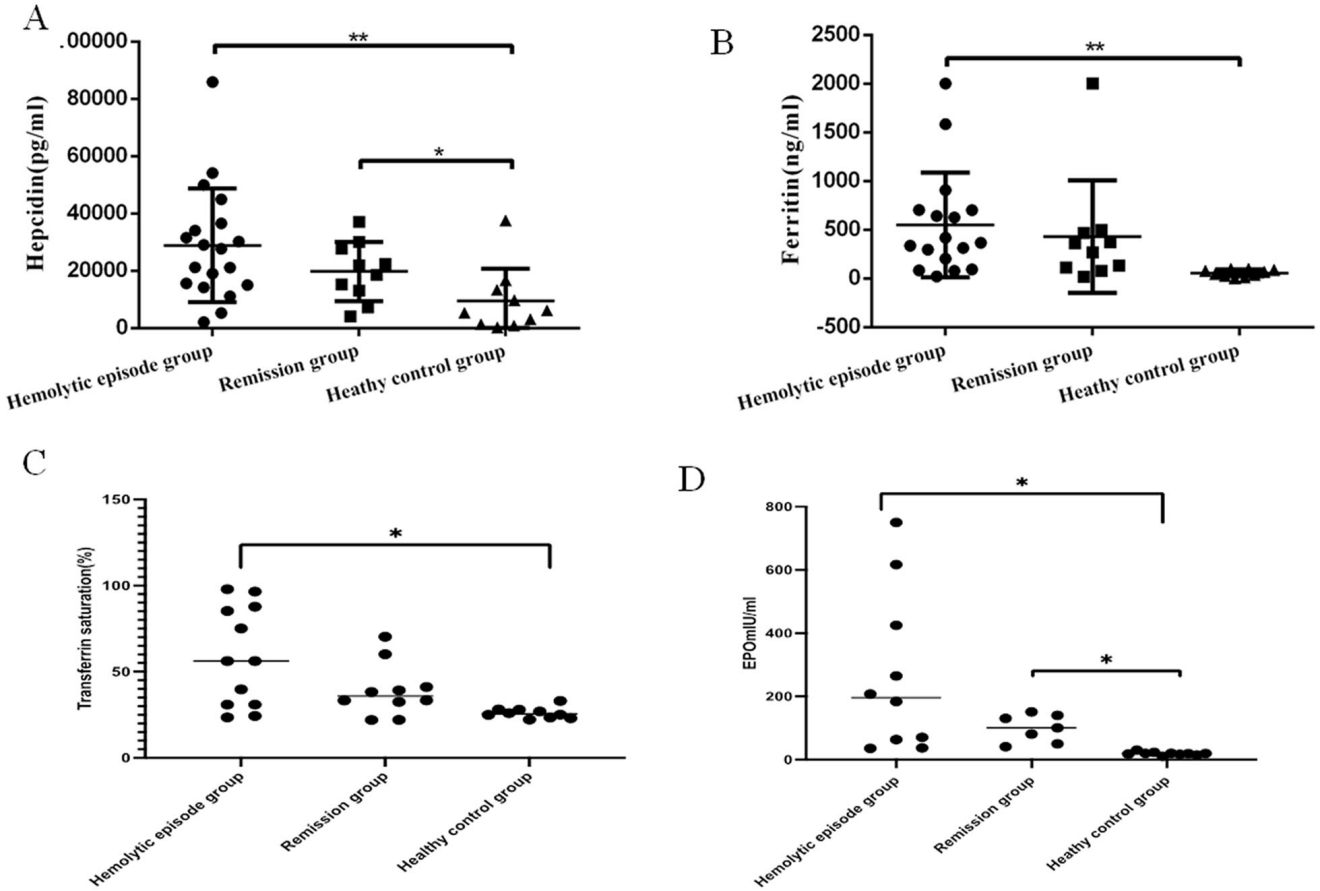
(A) The plasma level of hepcidin for hemolytic episode AIHA patients (28888 ± 4546 pg/ml, *p* = 0.0083) and remission AIHA patients (19766 ± 3250 pg/ml, *p* = 0.0473) were both obviously higher than that of normal control (9482 ± 3572 pg/ml). (B) The plasma level of ferritin for hemolytic episode AIHA patients (551.2 ± 130.3 ng/ml, *p* = 0.008) were significantly higher than that of normal control (56.91 ± 11.56) ng/ml. (C) Transferrin saturation was significantly higher in patients in the hemolytic episode group, [57.21 ± 8.96) %] than in healthy controls[ (30.23 ± 10.2) %, *p* = 0.013]. (D) EPO levels were higher in both the hemolytic group [(265.7 ± 80.08) mIU/ml] and the hemolysis remission group [(105.2 ± 30.1) mIU/ml, *p* = 0.021] than in healthy controls[(19.16 ± 5.33) mIU/ml, *p* = 0.018]. *p<0.05; **p<0.01

Serum level of hepcidin in hemolytic episode AIHA patients and remission patients were significantly higher than that in HCs (28,888 ± 4,546 and 19,766 ± 3,250 vs. 9,482 ± 3,572 pg/mL, respectively; *p* = 0.0083 and 0.0473 respectively). Ferritin levels was significantly higher in patients with hemolytic AIHA than that in HCs (551.2 ± 130.3 vs. 56.91 ± 11.56 ng/mL, *p* = 0.008). Transferrin saturation was significantly higher in the hemolytic episode group, [(7.21 ± 8.96)%] than in healthy controls[(30.23 ± 10.2)%, *p* = 0.013]. EPO levels were higher in both the hemolytic group [(265.7 ± 80.08) mIU/ml] and the remission group [(105.2 ± 30.1) mIU/ml, *p* = 0.021] than in healthy controls[(19.16 ± 5.33) mIU/ml, *p* = 0.018] (Figure 1).

**Figure 2.**
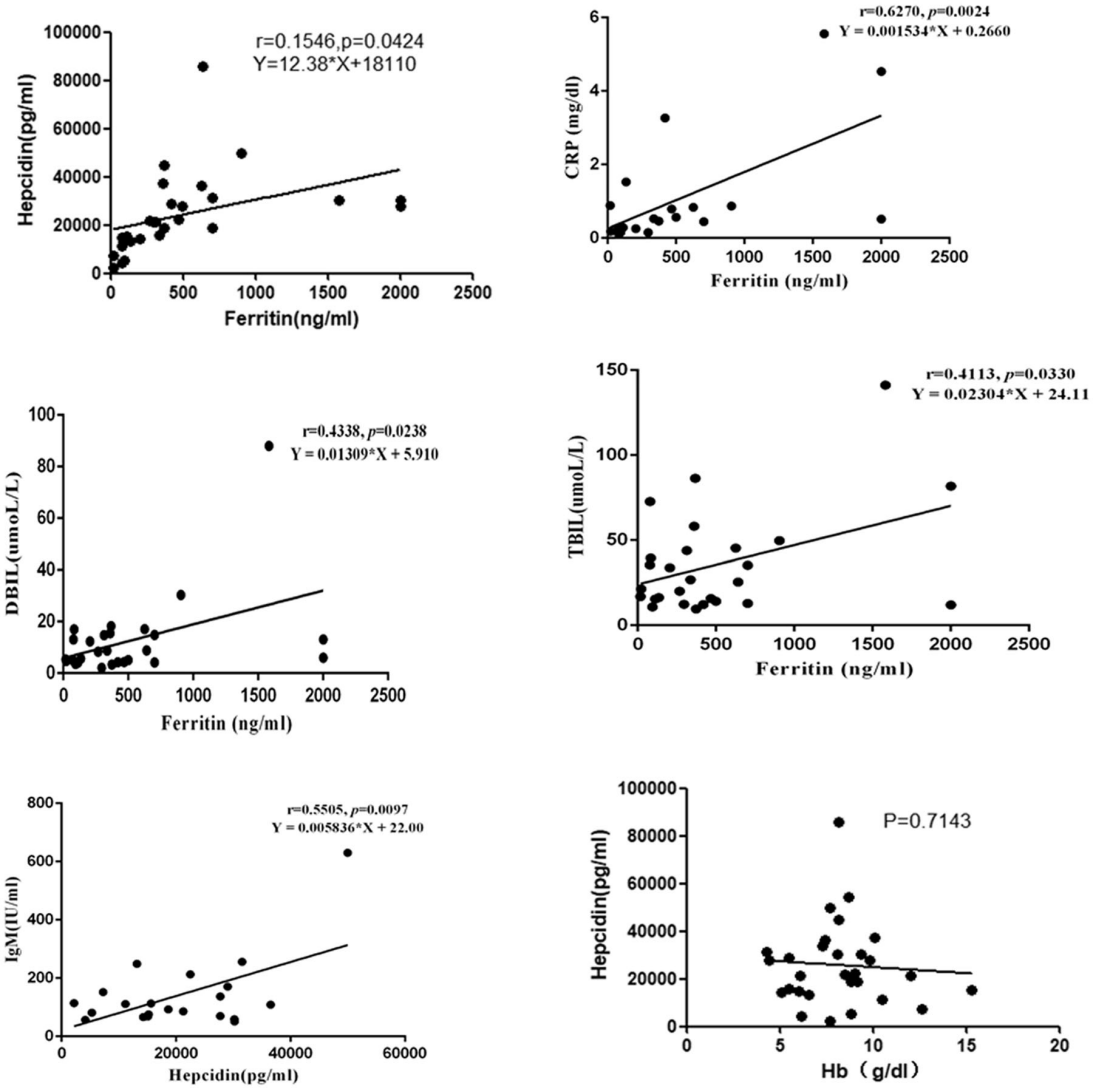
In AIHA patients, the level of IgM in peripheral blood was positively correlated with the plasma level of hepcidin (*r* = 0.5505, *p* = 0.0097) and the plasma level of TBIL, DBIL and CRP were positively correlated with the level of ferritin, respectively (*r* = 0.4113, *p* = 0.0330; *r* = 0.4338, *p* = 0.0238; *r* = 0.6270, *p* = 0.024). In AIHA patients, the level of hepcidin was positively correlated with the ferritin (*r* = 0.1546, *p* = 0.042), and but not with Hb, Ret, FHb and HP (*p* > 0.05).

In AIHA patients, the level of hepcidin was positively correlated with the ferritin (*r* = 0.1546, *p* = 0.042), and but not with Hb, Ret, FHb and HP (*p* > 0.05). Positive correlations were found between immunoglobulin M (IgM) and serum hepcidin (*r* = 0.5505, *p* = 0.0097), and between ferritin and the hemolytic indicators TBIL and DBIL (*r* = 0.4113, *p* = 0.0330; *r* = 0.4338, *p* = 0.0238), as well as the inflammatory biomarker CRP (*r* = 0.6270, *p* = 0.0024) in patients with AIHA (Figure 2).

### 3.3. Body weight, RBCs Hb, TBIL, IBIL and hepcidin findings in AIHA models

The model mice weighed significantly less than the control mice (20.42 ± 0.18 vs. 22.11 ± 0.34 g, *p* < 0.01). Values were significantly decreased in the models compared with control mice for RBCs (6.54 ± 0.09 vs. 9.36 ± 0.29 × 10^12^/L, *p* < 0.01) and Hb (10.22 ± 0.13 vs 12.8 ± 0.39 g/dL, *p* < 0.01). Values were significantly increased in the models compared with control mice for TBIL (16.12 ± 0.64 vs. 8.29 ± 0.68 μmol/L, *p* < 0.01), IBIL (13.3 ± 0.61 vs. 5.67 ± 0.7 μmol/L, *p* < 0.01), LDH (581.7 ± 26.14 vs. 215.4 ± 24.12 U/L, *p* < 0.01) and hepcidin (8,508 ± 733.7 vs. 4,459 ± 294.6 pg/mL, *p* < 0.05) (Table 3).

**Table 3.** The body weight, blood routine, biochemistry indicators and plasma level of hepcidin for two groups.

Group	AIHA murine models	Normal control group
(number)	(45)	(20)
Body weight (g)	20.42 ± 0.18**	22.11 ± 0.34
RBC (×1012/L)	6.54 ± 0.09**	9.36 ± 0.29
Hb (g/L)	102.2 ± 1.28**	128.0 ± 3.94
TBIL (umoL/L)	16.12 ± 0.64**	8.29 ± 0.68
DBIL (umoL/L)	2.82 ± 0.17	2.6 ± 0.24
IBIL (umoL/L)	13.30 ± 0.61**	5.7 ± 0.70
LDH (U/L)	581.7 ± 26.14**	215.4 ± 24.12
Hepcidin (pg/ml)	8508 ± 733.7*	4459 ± 294.6

Compared with normal control group, *p<0.05; **p<0.01.

### 3.4. Biochemical and hemolytic indicators in AIHA mouse models after *Jianpishengxue keli* intervention for 14 and 28 days

After 14 days of *Jianpishengxue keli* administered at three doses, values for the body weight, RBCs, and Hb were still significantly lower, whereas levels of hepcidin, serum TBIL, and LDH were significantly higher in the mouse models than in the normal and controls (*p* < 0.01 for all). Serum IBIL levels were higher in the models given low and medium doses of *Jianpishengxue keli* than in controls (*p* < 0.01), whereas IBIL did not significantly differ between models given the high dose and controls (Table 4).

**Table 4.** Body weight, blood routine, biochemistry indicators and plasma level of hepcidin for each experimental group 14 days after *Jianpishengxue Keli* administration.

	Normal control group	AIHA murine models	Low-dose group	Medium-dose group	High-dose group	AIHA with *Jianpishengxuekeli*
Body weight (g)	23.15 ± 0.30	20.64 ± 0.29**	20.83 ± 0.30**	20.50 ± 0.31**	21.00 ± 0.28**	20.90 ± 0.13^a^
RBC (×10^12^/L)	10.93 ± 0.31	8.19 ± 0.35**	8.22 ± 0.36**	8.49 ± 0.41**	8.7 ± 0.36**	8.68 ± 0.24
Hb (g/dL)	12.77 ± 0.36	10.30 ± 0.26**	10.29 ± 0.27**	10.42 ± 0.34**	10.62 ± 0.18**	10.56 ± 0.24
TBIL (umoL/L)	5.81 ± 0.50	12.87 ± 0.75**	12.82 ± 0.76**	9.89 ± 0.61**	8.18 ± 0.32**	9.05 ± 0.24^a^
DBIL (umoL/L)	1.97 ± 0.25	3.02 ± 0.31*	2.52 ± 0.39	2.52 ± 0.36	3.02 ± 0.43*	2.58 ± 0.26
IBIL (umoL/L)	3.84 ± 0.55	9.85 ± 0.59**	10.30 ± 0.74**	7.37 ± 0.82**	5.16 ± 0.45	6.17 ± 0.19^a^
LDH (U/L)	206.6 ± 19.02	531.1 ± 39.87**	552.8 ± 62.47**	526.8 ± 59.3**	330.8 ± 26.79**	380.8 ± 43.3
Hepcidin (pg/ml)	3198 ± 231	8107 ± 597.9**	7741 ± 541.8**	6483 ± 801**	5570 ± 519.7**	5770 ± 601^b^

Compared with normal control group, *p<0.05; **p<0.01, Compared with AIHA murine models on 0 day, ^a^*p*<0.05; ^b^*p*<0.01.

After 28 days of *Jianpishengxue keli* administration, the models given a low dose of *Jianpishengxue keli* weighed significantly less (*p* < 0.01), and had significantly higher serum LDH levels (*p* < 0.05) compared with the controls. Body weight and LDH did not significantly differ between the models given medium or high doses of *Jianpishengxue keli* and controls. Values for RBCs and Hb were lower, and serum hepcidin levels were higher in the models given low and medium doses of *Jianpishengxue keli* than in controls (*p* < 0.05 for all). However, values for RBCs, Hb, and hepcidin did not significantly differ between the high-dose and control groups. Serum TBIL and IBIL levels did not significantly differ between the experimental and control groups (Table 4).

### 3.5. *Jianpishengxue keli* for 14 and 28 days significantly decreased expression of hepatic hepcidin mRNA

Significantly more hepatic hepcidin mRNA was expressed in the AIHA mouse models than in controls (20.15 ± 1.449vs.1.089 ± 0.144, *p* < 0.01). After 14 days, hepatic hepcidin mRNA levels in models without and with low, medium and high doses of *Jianpishengxue keli* were all still significantly higher than that in the controls (18.70 ± 0.715, 13.78 ± 1.355, 13.85 ± 1.968 and 9.161 ± 0.941 vs. 1.011 ± 0.117, all *p* < 0.01). However, the hepatic hepcidin mRNA was significantly lower after, than before intervention with low, medium, and high doses of *Jianpishengxue keli* (13.78 ± 1.355, 13.85 ± 1.968 and 9.161 ± 0.941 vs. 20.15 ± 1.449; *p* < 0.05, 0.05, and 0.01, respectively). Hepatic hepcidin mRNA levels did not significantly differ in the models without drug intervention compared with before (18.70 ± 0.715 vs. 20.15 ± 1.449) (Table 5).

**Table 5.** The expression level of hepatic hepcidin mRNA of each experimental group.

	Normal control group	AIHA murine models	Low-dose group	Medium-dose group	High-dose group
0 Day	1.089 ± 0.144	20.15 ± 1.449**			
14 Days	1.011 ± 0.117	18.70 ± 0.715**	13.78 ± 1.355^**a^	13.85 ± 1.968^**a^	9.161 ± 0.941^**b^
28 Days	1.161 ± 0.306	12.42 ± 1.026^**a^	6.6 ± 0.873^**b^	5.511 ± 0.517^**b^	2.816 ± 0.4^*b^

Compared with normal control group, *p<0.05; **p<0.01; compared with AIHA murine models on 0 day, ^a^*p*<0.05; ^b^*p*<0.01.

After 28 days of administration, hepatic hepcidin mRNA levels were significantly higher in the models (12.42 ± 1.026, *p* < 0.01), and in the models given low, medium, and high doses of *Jianpishengxue keli* compared with controls (6.6 ± 0.873, 5.511 ± 0.517, and 2.816 ± 0.4 vs. 1.161 ± 0.306; *p* < 0.01, <0.01 and < 0.05, respectively). However, hepatic hepcidin mRNA levels in the models without (12.42 ± 1.026, *p* < 0.05), and with low, medium and high doses of *Jianpishengxue keli* were significantly lower after, than before intervention (6.6 ± 0.873, 5.511 ± 0.517, and 2.816 ± 0.4 vs. 20.15 ± 1.449; *p* < 0.05 for all) (Table 6).

**Table 6.** Body weight, blood routine, biochemistry indicators and plasma level of hepcidin for each experimental group 28 days after *Jianpishengxue Keli* administration.

	Normal control group	AIHA murine models	Low-dose group	Medium-dose group	High-dose group	AIHA with *Jianpishengxuekeli*
Body weight (g)	23.32 ± 0.33	20.52 ± 0.43**	20.04 ± 0.52**	23.22 ± 0.34	23.26 ± 0.42	22.17 ± 0.18^b^
RBC (×10^12^/L)	11.36 ± 0.32	8.93 ± 0.50**	9.51 ± 0.34**	9.33 ± 0.42**	10.80 ± 0.23	9.88 ± 0.25
Hb (g/dL)	13.44 ± 0.58	10.36 ± 0.23**	10.00 ± 0.21**	11.42 ± 0.42*	12.46 ± 0.65	11.29 ± 0.36
TBIL (umoL/L)	3.62 ± 0.39	6.5 ± 0.51**	4.15 ± 0.55	3.63 ± 0.46	3.44 ± 0.42	3.74 ± 0.27^b^
DBIL (umoL/L)	1.67 ± 0.17	1.72 ± 0.39	1.71 ± 0.30	1.73 ± 0.43	1.98 ± 0.38	1.81 ± 0.20
IBIL (umoL/L)	1.95 ± 0.38	4.79 ± 0.66**	2.43 ± 0.48	1.9 ± 0.19	1.47 ± 0.41	1.94 ± 0.23^b^
LDH (U/L)	205.7 ± 30.45	408.4 ± 69.97*	419.4 ± 74.34*	310.6 ± 56.22	177.0 ± 43.28	302.3 ± 41.32
Hepcidin (pg/ml)	3014 ± 455.7	7514 ± 893.6**	7060 ± 533.6**	5446 ± 635.6*	3751 ± 364.7	5419 ± 456.7^a^

Compared with normal control group, *p<0.05; **p<0.01. Compared with AIHA murine models on 0 day, ^a^*p*<0.05; ^b^*p*<0.01.

### 3.6. Hepatic FPN1 levels after *Jianpishengxue keli* administration 14 and 28 days

**Figure 3.**
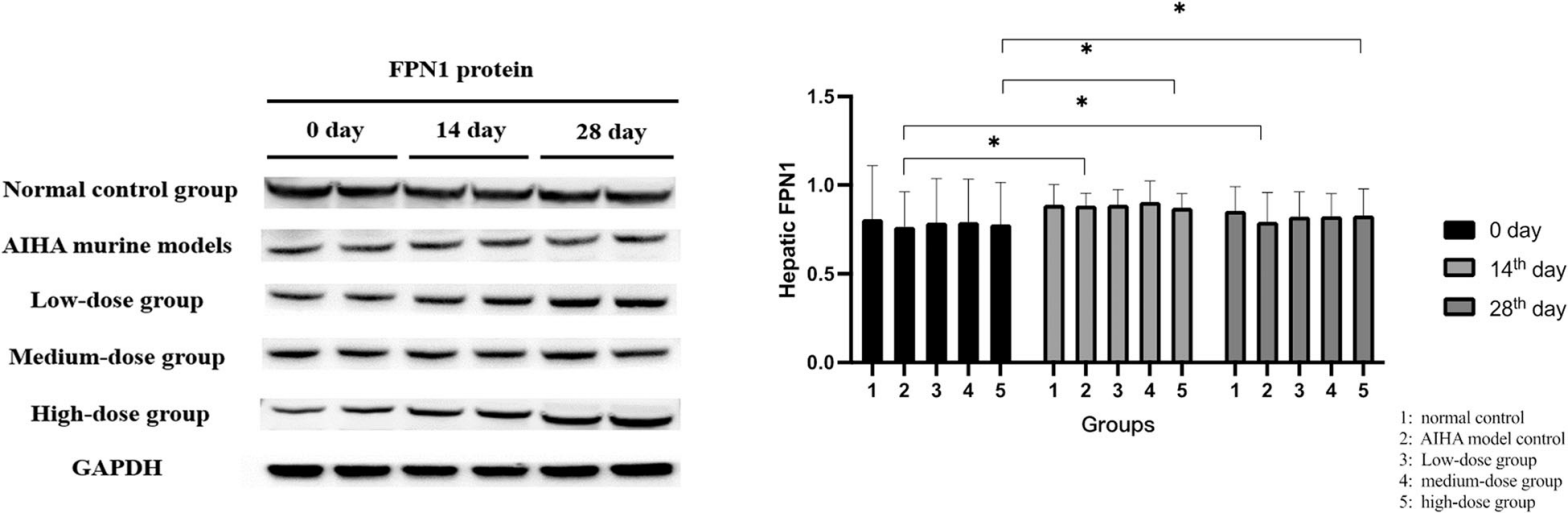
The expression of hepatic FPN1 protein. Hepatic FPN1 protein (62 kDa) were significantly lower in mouse models of AIHA than in the normal controls, but these increased in the models given any of the three doses of *Jianpishengxue keli* for 14 and 28 days. *p < 0.05.

Hepatic FPN1 protein (62 kDa) were significantly lower in AIHA mouse models than in the normal controls, but these increased in the models given any of the three doses of *Jianpishengxue keli* for 14 and 28 days. Hepatic FPN1 protein levels in the high-dose group significantly increased after 28 days of administration (Figure 3).

## 4. Discussion

Autoimmune hemolytic anemia (AIHA) is characterized by hemolysis, i.e. the breakdown of red blood cells (RBCs) which occurs with autoantibodies and/or complement, together with activated macrophages, T-lymphocytes and cytokines all contributing to the process [23]. Patients with AIHA often reach a state of chronic immunological inflammation due to the prolonged course of the disease and recurrent hemolytic attacks [9].

We found significantly higher serum hepcidin and ferritin levels in patients with AIHA than in healthy controls. And it was confirmed that serum hepcidin levels positively correlated with ferritin and CRP levels. Levels of IgM positively correlated with serum hepcidin. TBIL, DBIL, IgG and IgA did not correlate with serum hepcidin. It suggested that there is inflammatory reaction and abnormal iron metabolism during AIHA hemolysis. The AIHA mouse model was successfully constructed by injecting rat red blood cells [20,21]. The weight of the mouse model decreased, TBIL and IBIL increased significantly, and LDH was much higher than that of the control mice. Ferritin and hepcidin levels were found to be significantly higher in the AIHA mouse model, significantly increased serum hepcidin and hepatic hepcidin mRNA expression in the models were consistent with the findings of patients with AIHA, suggesting that an inflammatory response, disordered iron metabolism in AIHA. During our literature review for an association between AIHA and iron overload, we found only a single case of iron overload in a patient with Evans syndrome presenting with a severe AIHA crisis requiring blood transfusion of 20 units [24]. Only two patients in this group required transfusion of red blood cells due to their condition, and the majority of patients did not receive blood transfusion during the consultation, and the increase in ferritin was not related to the amount of blood transfusion.

Hepcidin is a key hormone that regulates iron metabolism, controls iron absorption in the intestine, and regulates iron homeostasis and affects iron recycling [25]. Hepcidin regulates iron levels through interaction with its downstream target Ferroportin 1(FPN1) which is a transmembrane iron-export protein that is expressed on the cell surfaces of most tissues and is the only known cellular iron release pathway. Hepcidin can reduce iron absorption in the intestine by binding to FPN1 and promoting its internalization and degradation in lysosomes. It can also decrease circulating iron levels by reducing iron release from the liver and the reticuloendothelial system [26,27]. Hepcidin not only regulates iron metabolism, but also affects erythropoiesis and it has been identified to hinder effective erythropoiesis [28]. Inflammatory factors, such as interleukin-6 (IL-6), can induce hepcidin gene expression in the liver [29]. Long-term stimulation of the chronic inflammatory response can promote the upregulated and downregulated expression of hepcidin and FPN1, respectively, in the liver. Upregulated erythropoietin (EPO) can inhibit hepcidin mRNA under conditions such as hypoxia or an iron deficiency [30].

In response to erythropoietin (EPO) stimulation, Erythroferrone (ERFE), produced by erythroblasts, is an erythroid regulator and acts on the liver to decrease hepcidin production by inhibiting bone morphogenetic protein (BMP)-small mothers against decapentaplegic (SMAD) pathway [31]. Fibroblast growth factor 23 (FGF23) has been shown to positively influence hepcidin concentration [32]. Notably, EPO-ERFE and FGF 23 act bi-directionally as important regulators of each other [33]. In animal models, ERFE has been proposed to contribute to recovery from anemia of inflammation by suppressing hepcidin [34]. Liu Xu [35] found that the expression level of ERFE in 12 AIHA patients was higher than that in the normal control group. In this group the serum EPO level of AIHA patients was higher than that of healthy control. The association and impact of EPO and Hepcidin in AIHA needs to be studied in depth.

There is no licensed treatment for autoimmune hemolytic anemias (AIHAs), although some national guidelines do exist. Glucocorticoid is the first-line treatment for AIHA, but rates of recurrence are high and that of complete remission is relatively low, and the disease course can become chronic [36,37]. Traditional Chinese and Western medicines have gradually become integrated in treatment strategies for the diagnosis and treatment of various diseases. *Jianpishengxue keli* is a compound preparation that includes *Codonopsis pilosula*, *Poria cocos*, *Atractylodes*, licorice, *Astragalus*, Chinese yam, Chicken gizzard membrane, tortoise shell, *Ophiopogon japonicus*, *Kadsura longepedunculata*, oysters, *Ziziphus jujuba*, ferrous sulfate and vitamin C. Each gram of extract is equivalent to 3.3 g of raw medicinal materials, containing 0.154 g of FeSO4•7H_2_O [11].

IBIL was significantly decreased in model mice administered with a high dose of *Jianpishengxue keli* for 14 days, suggesting that *Jianpishengxue keli* can inhibit hemolysis. In addition, RBCs, Hb, and hepcidin completely recovered in the high-dose group. These results showed that low-dose *Jianpishengxue keli* improved the hemolytic status, and that high-dose *Jianpishengxue keli* rapidly improved the hemolytic status of the model mice, promoted the recovery of hematopoietic function. Although hepatic hepcidin mRNA remained significantly higher after 28 days of intervention in the high-dose group, it was significantly lower than that before the intervention. Hepatic FPN1 protein levels were significantly lower in model mice than in controls. Hepatic FPN1 protein expression was significantly increased in the high-dose group at 28 days of intervention, indicating the recovery of iron metabolism and a damped immune inflammatory response.

## 5. Conclusion

AIHA patients have abnormal iron metabolism. *Jianpishengxue keli* can improve the general condition and hemolysis in AIHA mouse model. But the specific mechanism and pathway of *Jianpishengxue keli* in AIHA need to be further studied.

## Data Availability

All the data used to support the findings of this study are available from corresponding authors upon reasonable request.
